# Serotonin—A Driver of Progressive Heart Valve Disease

**DOI:** 10.3389/fcvm.2022.774573

**Published:** 2022-01-28

**Authors:** Helge Waldum, Alexander Wahba

**Affiliations:** ^1^Department of Clinical and Molecular Medicine, Faculty of Medicine and Health Sciences, Norwegian University of Science and Technology, Trondheim, Norway; ^2^Department of Cardio-Thoracic Surgery, St Olav's University Hospital, Trondheim, Norway; ^3^Department of Circulation and Medical Imaging, Norwegian University of Science and Technology, Trondheim, Norway

**Keywords:** serotonin, heart valve, platelets, serotonin receptor 2B, flow disturbance

## Abstract

It is well known that some serotoninergic drugs and neuroendocrine tumors producing serotonin (5-HT) may induce valvular heart disease by stimulation of proliferation of valvular cells *via* interaction with a 5-HT receptor type 2B. Serotonin could play a role in the pathogenesis of progressive valvular disease for example as a complication of rheumatic fever, in patients with congenital bicuspid aortic valves or in degenerative aortic valve stenosis. The initial inflammation in acute rheumatic fever seems to affect both right and the left-side cardiac valves. Some patients develop chronic right-sided valve disease, particularly in connection with septum defects, though left-sided valves typically are predominantly affected, indicating that high flow velocity and systemic pressure close to the valves may be central in the pathogenesis. Serotonin is transported in granules in blood platelets. Changes in platelet number and concentrations of substances released from platelets in patients with valvular disease indicate that serotonin is released locally by shear stress when passing through an abnormal valve. Accordingly, any functional changes (like bicuspid aortic valves and changes secondary to degeneration) in the valves may progress due to locally released serotonin. Unfortunately, due to serotonin release by sampling and preparation of plasma, local serotonin assessment is not possible. Nevertheless, we suggest that serotonin may play a role in valvular disease in general and that patients may benefit from treatment reducing the effect of serotonin on the heart.

## Introduction

Heart valve disturbances of function like stenosis or insufficiency are serious conditions that may lead to the development of heart failure if untreated. Valve replacement by open heart surgery or by percutaneous catheter technique represents the available therapeutic options. No pharmacological treatments have proved efficacious to date.

A century ago, acute rheumatic fever was the major cause of valvular heart disease, but during the last decades, this disease has more or less disappeared from the Western world (1). Other entities, such as congenital bicuspid aortic valves, are more prominent causes of aortic valve stenosis (2). Moreover, the destruction of valves at the right side of the heart, due to small bowel neuroendocrine tumor (NET) (3), is well known. In this short review, we argue that serotonin may be a shared, important and poorly appreciated factor in the progression of valvular heart stenosis of different etiologies.

## Rheumatic Heart Disease Following Acute Rheumatic Fever (ARF)

ARF is caused by streptococcal infection usually starting in the pharynx (4). The mechanism by which streptococcal infection leads to affection of the heart and also the brain causing chorea, is not completely clear, but similarities between antigens at affected structures and the specific strains of streptococci, have been more or less accepted (5). Nevertheless, there is no indication of a direct bacterial infection of the heart nor brain. The patient may overcome the causative streptococcus by developing immunity or with antibiotics. In the acute phase of the infection or with later reinfections, there is inflammation of affected organs such as the heart and the brain. The use of echocardiography allows detection of subclinical valve inflammation during the acute phase (6) and may also be used in follow-up. The inflammation dwindles when the streptococcus is eradicated, and patient becomes asymptomatic. After decades, cardiac symptoms may reappear due to progressive destruction of cardiac valves, most often on the left side of the heart that is exposed to systemic pressure (7). Interestingly, the right sided valves exposed to lower pressure seldom develop chronic changes. However, septal defects resulting in increased pressure even in the right chambers of the heart, are more prone to valvular changes (8). This suggests that all valves of the heart are involved in the initial rheumatic inflammation. An important role of flow changes with shear stress may thus be of importance for the progression of valvular changes (9). We postulate, that by some way or other, the streptococcus causing acute rheumatic fever triggers an inflammation in the heart valves that with exposure to systemic pressure and flow changes may result in a progressive valve destruction. The lack of progressive chronic changes of right-sided heart valves suggests that the mechanism inducing long-term left-sided valvopathy is different from the inflammation related to the initial streptococcal infection. A review from 2016 concluded that secondary penicillin prophylaxis for prevention of recurrent rheumatic fever was the only possible treatment to reduce the risk of progressive rheumatic heart disease (4).

## Aortic Stenosis in Congenital Bicuspid Aortic Valve

Up to 2% of children are born with bicuspid aortic valve, which may lead to a progressive valvular disease later in life (2). There are no signs of inflammation or other changes in these valves at birth. Nevertheless, bicuspid valves predispose to development of aortic stenosis (2), thus demonstrating that flow disturbance alone (10) may induce valvular disease without any pre-existing inflammation.

Accordingly, it could be postulated that flow changes alone can lead to valvular disease in bicuspid aortic valves, whereas in rheumatic valvular disease, systemic pressure and flow disturbances are a prerequisite for progressive valvular damage following the initial structural changes induced by the pharyngeal streptococcal infection.

## Neuroendocrine Tumor (Previously Carcinoid) Heart Disease

It is well known that NETs from the small intestine with liver metastasis may induce valvular heart disease. Usually, the valves on the right side of the heart are affected. However, also left-sided cardiac disease may occur (11). In these patients there was no pre-existing valvular inflammation nor valvopathy, and the involvement of the right-sided valves on the low-pressure side of the heart, shows that systemic pressure is not necessary to cause progressive valvular disease. The small intestinal NETs develop from the enterochromaffin (EC) cell which quantitatively is the major producer of serotonin in the body (12). Serotonin is transported in the blood by platelets after serotonin uptake in megakaryocytes as well as the platelets themselves. In small intestine NETs without liver metastasis, serotonin is metabolized in the liver into biologically inactive compounds. Once liver metastasis occurs, serotonin reaches the right side of the heart, before mainly being metabolized in the lungs. Although EC cell NETs starting outside the portal system may induce systemic symptoms like flushing before having metastasised, valvular heart disease is rarely encountered in these cases. The reason may be that these patients are diagnosed at an earlier phase due to early typical symptoms such as flushing. In patients with small intestinal NETs fibrosis is not limited to heart valves but occurs in other areas, including locally as mesenteric desmoplasia, presumably also due to serotonin (13).

### Serotonin Receptors Involved

We have shown that long-term subcutaneously administered serotonin to rats, induced valvular heart disease *via* trophic (14) stimulation of subendothelial cells by interaction with a serotonin 2B receptor (15). There are many different types of serotonin receptors, and receptors of type 2B have been shown to be central in heart development and probably also play an important role in adult life (16). We showed that the 5HT(2B/2C) antagonist terguride prevented the valvular changes induced by serotonin in the rat (17). However, in publication on cultures of sheep aortic valve interstitial cells (SAVIC), exposed to different serotonin agonists or antagonists, the authors concluded that the receptor mediating the serotonin signaling pathway was of the 5HT(2A) type (18). In humans, the valvular cell receptor 5HT(2B) was demonstrated by real time PCR (19). There is a large homology between 5HTR types 2A and 2B (20) which, together with species differences, can make the classification difficult. Receptors of type 5HT(2B) also play a role in pulmonal hypertension (21). It is now generally accepted that the most likely serotonergic receptor transmitting the fibrosis of the valves is the 5-HT(2B) type (22). Serotoninergic drugs have also been known to induce valvular heart disease in man (23) as well as fibrosis in other tissues (24). Thus, it is well documented that serotonin can cause valvular heart disease. In a comparison between normal human valves and pathological valves, secondary to NETs (carcinoids), nodules of smooth muscle actin were detected in the interstitial cells (25). Furthermore, TGF (transforming growth factor)-β, the factor believed to transmit serotonin induced fibrosis, was expressed (25), Serotonin also stimulated Erk in SAVIC cells (18).

### Serotonin in Blood

**Figure 1:**
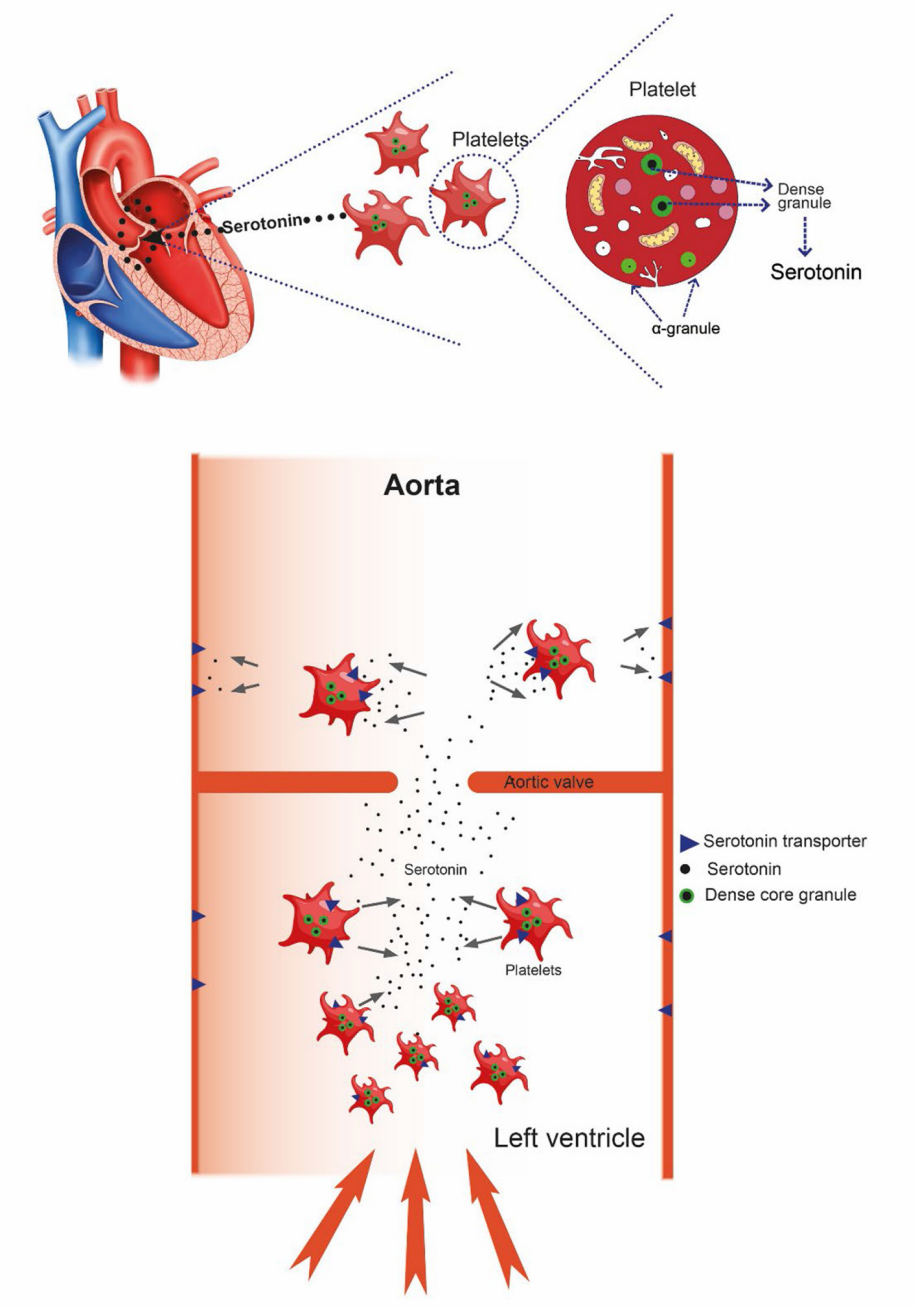
With increased flow velocity and pressure at the aortic valve, platelets may be damaged causing release of serotonin resulting in progressive damage to the valve itself. After passing the valve, serotonin is taken up by the serotonin transporter (SERT), restoring very low serotonin level.

Due to release of serotonin from platelets during blood sampling and plasma preparation, free blood serotonin concentration is not known. However, as written in a previous review, it is presumed to be extremely low (26). An important reason for the low concentration of serotonin is the serotonin transporter (SERT) which pumps serotonin into the cells. It is expressed on platelets and endothelial cells (27) (Figure 1). Interestingly, deficiency of the 5-hydroxytryptamine transporter gene causes valvopathy in mice (28), thus demonstrating the importance of keeping the serum serotonin concentration low. Moreover, the serotonin receptor type 2B has very recently been reported to mediate mineralization in murine valvular interstitial cells (29), and activated platelets were recently reported to promote an osteogenic programme and the progression of calcific aortic valve stenosis (30). Calcification of heart valves is naturally an important factor in loss of valve function (31) and is presumably irreversible. To conclude this section, serotonin alone can induce all phases of valvular disease.

## The Role of Serotonin in Valvular Heart Disease Due to Disturbance of Flow

Serotonin is mainly produced in EC cell and transported in the blood by platelets where it is stored in specific granules (32). These granules are degranulated when exposed to damaged endothelium or probably also due to physical strain imposed by flow changes (33). It is well known that red blood cell may be injured in patients with aortic stenosis, leading to anemia (34). Also, platelets may be affected since such patients also may have thrombocytopenia (35). Platelet function assessed *in vitro* by closure time analysis has been found to be prolonged in a high proportion of patients with aortic stenosis (36). A reduction in high molecular weight Von Willebrand factor also reflects the high shear stress in aortic stenosis (36). Reduced platelet function leading to bleeding tendency is well-known in patients with aortic stenosis (9, 37). Interestingly, platelet derived serotonin was reported to be responsible for fibrosis secondary to vascular disease (38). We do not know of any study where serotonin in blood from the left ventricle in patients with aortic stenosis has been measured. Unfortunately, blood sampling itself as well as the separation of blood corpuscles from the plasma induce release of serotonin from the platelets, masking possible low differences in serotonin concentration in the heart (39, 40). Due to the uncertainties in assessing serotonin in blood, it is not established that free serotonin circulates or its half-life in blood. Thus, serotonin should not be regarded as a hormone, but instead a potent and possibly harmful signaling substance, which is transported in platelet granules and released upon need. When exposed to shear stress, due to disturbances of flow near valves, damage to the platelets may result in local release of serotonin. Of importance is also the association between idiopathic thrombocytopenic purpura and aortic valve disease (41). Over time, through the trophic effects on some cell types in the valves, serotonin may damage the valve function. Even without previous known malfunction of the valves, minor degenerative changes may be present which in old age could predispose to for instance calcific aortic valve disease (CAVD). Atherosclerosis has been incriminated in the pathogenesis of CAVD although there are also marked differences (42). Of unknown reason, the degree of calcification is markedly higher in males compared with females (43). As previously mentioned, serotonin may also induce calcification in murine valvular cells (29) and affect calcification in pig valvular cells *in vitro* (44). Furthermore, in a scanning electron microscope study on valves from mice and man, the authors concluded that their findings suggested a role for platelets in the progression of CAVD (30). In recent years genetic abnormalities, particularly in reduced Notch1 expression, have been regarded as a possible mechanism for calcification in CAVD (45). Interestingly, genetic ablation of the 5HT receptor 2B improved aortic valve hemodynamics of Notch1 heterozygous mice fed high-cholesterol diet (46). Actually, no effective pharmacologic treatment of CAVD has been found (42, 47). Of great interest are two recent papers describing effects of serotonin not only on aortic valves, but also mitral valves (44, 48). Therefore, serotonin may be involved in the pathogenesis not only in aortic valvular disease, but also in mitral valvular myxomatous degeneration which is the second most prevalent valvular heart disease.

### Summing-Up and Future Perspectives

We therefore hypothesize that serotonin may be an important driver of aortic stenosis in particular, but also heart valvular disease in general since; (1) serotonin alone is capable to induce valvular changes in the heart; (2) serotonergic drugs and tumors producing serotonin do induce such changes; (3) congenital valvular changes without a pre-existing inflammation leading to flow changes and probable platelet release of serotonin; and (4) an initial inflammation of valves may progress to chronic valvular disease mainly in valves localized to high pressure chambers (Table 1); (5) animal and *in vitro* basic studies have shown that serotonin is involved in mechanisms that probably are involved in fibrosis and calcification. A general role of serotonin in cardiac valve degeneration or disease was also suggested in two relatively recent reviews (49, 50). We believe the role of serotonin in progressive valvular heart disease (Table 1) warrants further investigation. We have tried to establish an aortic stenosis model in rats, but without success. Studies in larger animals could perhaps solve the problem. Importantly, a rat study of serotonin injections for 12 weeks controlled by echocardiography and by histopathology showed thickened valves with functional valvular defects. Eight weeks without serotonin- dosing normalized the changes (51). This reversibility suggests that also in humans antagonist treatment at a relatively early phase not only may stop progression, but also reverse prior valvular damage. It is possible that serotonin released from platelets at abnormalities in the arterial tree could play a role in the progress of the changes.

**Table 1:** Serotonin and heart valve disease.

**Species**	**Disease/condition**	**Heart affection**		**Inflammation**	**Platelet changes**	**References**
		**Left sided**	**Right sided**			
Rat	Subcutaneus injections	X	X			(14)
Man	Serotonergic drugs	X				(18)
Man	EC cell carcinoid (NET)	(X)	X			(10)
Man	Congenit changes including bicuspid aortic valves	X			X	(2, 22, 23)
Man	Acute rheumatic fever	X	(X)	Initially	X	(8, 9)
Man	Calcific aortic valve disease	X			X	(29)

## Conclusion

The serotonin receptor transmitting the changes in the valves is 5HT(2B) (22), and a specific antagonist could possibly be used in the prevention of progressive valvular disease, eventually in combination with a 5HT synthesis inhibitor such as telotristat (52). The new aspect of this review is the focus on local platelet release of serotonin in the vicinity of the valves as a possible driving force of valvular disease. Clinical studies on patients with valvular disease with 5HT(2B) receptor antagonists will be needed to confirm our hypothesis.

## Author Contributions

HW took the initiative to this manuscript. Both authors have collaborated in the attempt to develop an animal model of aortic stenosis, and both have contributed to the writing of this manuscript.

## Conflict of Interest

The authors declare that the research was conducted in the absence of any commercial or financial relationships that could be construed as a potential conflict of interest.

## Publisher's Note

All claims expressed in this article are solely those of the authors and do not necessarily represent those of their affiliated organizations, or those of the publisher, the editors and the reviewers. Any product that may be evaluated in this article, or claim that may be made by its manufacturer, is not guaranteed or endorsed by the publisher.
